# Ambient Air VOC Levels in an Industrial Area of Turkey: Levels, Spatial Distribution, and Health Risk Assessment

**DOI:** 10.3390/toxics13070540

**Published:** 2025-06-27

**Authors:** Aykan Karademir, Ercan Arpaz, Şenay Çetin Doğruparmak, Gülsün Özgül

**Affiliations:** 1 Department of Environmental Engineering, Faculty of Engineering, Kocaeli University, 41001 Kocaeli, Turkey; aykan@kocaeli.edu.tr (A.K.); earpaz@kocaeli.edu.tr (E.A.); 2 Environmental Protection Technologies, Environmental Protection and Control Program, Kocaeli University Izmit Vocational School, 41285 Kocaeli, Turkey; gulsun@kocaeli.edu.tr

**Keywords:** volatile organic compounds, passive sampling, spatial distribution, health risk assessment, Dilovası, Turkey

## Abstract

The seasonal variations, spatial distribution, and health risk assessment of 13 volatile organic compounds (VOCs), particularly benzene, toluene, ethyl benzene, and xylene (BTEX), in the ambient air of Dilovası, a Turkish city with unplanned urbanization, are presented in this study. Using passive tube sampling, at 22 locations in Dilovası, air samples were collected separately for the summer and winter, and concentrations were measured using thermal desorption GC-MS. Pollution maps were created using the Golden Software Surfer program and QGIS Desktop 3.42.0 software program. A health risk evaluation was conducted using the US Environmental Protection Agency’s (USEPA) approach. The study’s findings demonstrated that the atmospheric VOC concentrations at the sampling locations varied significantly by season and location. According to a carcinogenic risk assessment, residents in this area may be more susceptible to cancer if they are exposed to benzene, ethylbenzene, and naphthalene over an extended period. A non-carcinogenic risk (HQ) evaluation determined that while there was no significant risk at 21 measurement points, there was a substantial risk for non-cancer health effects at 1 measurement point. The significance of regulatory policies and pollution control technologies has once again emerged in this context.

## 1. Introduction

As the population living in cities has increased rapidly in recent years, urban air pollution from residential heating, industrial activities, and vehicle traffic has become a significant problem, causing the deterioration of the quality of life and creating public health risks. Pollution-related problems are of interest, especially in the urban areas of developing countries, as rapid urbanization, poorly managed transportation systems, and irregular industrialization lead to rising levels of air pollution in populated regions and create pollution hotspots [1]. Therefore, many studies have been conducted to assess the sources, levels, trends, and health effects of pollutants in urban areas [2,3,4].

Pollutants of concern in urban areas include traffic- and industry-related volatile organic compounds (VOCs), which have attracted global attention over the past decade due to their inhalation toxicity and potential for secondary atmospheric reactions. Several VOCs, particularly benzene, toluene, ethylbenzene, and xylenes (BTEX), are classified as hazardous air pollutants (HAPs) by the United States Environmental Protection Agency (US EPA) [2]. Prolonged exposure to HAPs can cause major health issues. Benzene, for example, is a hematotoxic chemical that can cause myeloid leukemia and aplastic anemia, and poses risks to the lymphatic, central nervous, and blood-forming systems. Furthermore, maternal exposure to high concentrations of benzene can cause neural tube defects in infants [5,6]. Benzene is the most toxic chemical in the BTEX family [7]. According to the International Agency for Research on Cancer (IARC), benzene is classified as a Group 1 carcinogen, indicating sufficient evidence of carcinogenicity in humans [6]. Toluene, ethylbenzene, and xylenes are neurotoxic chemicals that have been associated with brain diseases, eye irritation, skin inflammation, premature birth, and issues with the respiratory system, liver, and kidneys. The risk levels and types of linked disorders differ based on the specific VOCs [2]. Therefore, monitoring BTEX in the lower atmosphere has become a priority in recent years for both human and ecosystem health [1].

The assessment of ambient VOC concentrations and their impact on health at the local, regional, and national levels has been the subject of numerous research studies in the literature. Studies carried out in urban areas have demonstrated that there is seasonal change in VOC levels and that these levels are influenced by a number of factors, including industry, transportation, and home heating [8,9,10,11,12]. For example, Xiong et al. assessed major sources of VOCs and associated health risks in eight Canadian cities from 2013 to 2018, based on ground observations from the national Air Pollution Surveillance network. They found that in Western Canada, the cancer risk from inhalation of VOCs was significantly above acceptable risk levels. Traffic locations were the most dangerous to human health, followed by industrial areas, urban centers, and background areas [9]. García-Garcinuño et al. evaluated 180 samples collected in 2023 via the passive monitoring of volatile organic compounds (VOCs) and semi-volatile organic compounds (SVOCs) from six metropolitan neighborhoods near Tarragona, one of Europe’s main petrochemical complexes. The risk assessment results found that benzene and 1,3-butadiene posed most of the risk [10]. In a study conducted by Sari and Bayram in 2014, VOC emissions from home heating systems in Izmir and the effects of these emissions on air quality were examined. It was found that VOC levels increased during the winter months, especially in residential areas in the city center, and this increase negatively affected air quality [11]. In 2011, Demir et al. evaluated 45 VOC chemicals at Istanbul’s Davutpasa Campus. Toluene was identified as the chemical with the highest concentration. The species’ daytime and nighttime concentration rates were also evaluated, and VOCs of industrial origin were found to have the highest rates, whereas biogenic VOCs had relatively stable and low concentrations [12].

Dilovası, a region in Turkey known for its significant environmental challenges due to unplanned urbanization and industrialization, has also been the subject of similar studies in previous years [13,14,15,16,17,18,19,20,21,22]. It is situated on the Marmara Sea coast between the metropolitan cities of Istanbul and Kocaeli. Due to its topographic conditions, the region is lower than the surrounding areas and has a bowl-shaped territory. This condition has a negative impact on the dilution of pollutants discharged into the atmosphere by vehicles and industrial sites. Because of this, the district is affected by nearly every kind of environmental pollution. Monitoring VOCs, particularly BTEX, has become even more crucial in the Dilovası district in the current climate, due to the district’s growing population, which has grown from about 44,000 in the 2010s to 55,000 today [23]. Parallel to population growth, traffic has also intensified, and people have spent more time driving from work to home. Recently, it has been reported that cancer has become the main cause of death in the region [14]. Therefore, monitoring the district’s air quality parameters is extremely beneficial.

**Figure 1:**
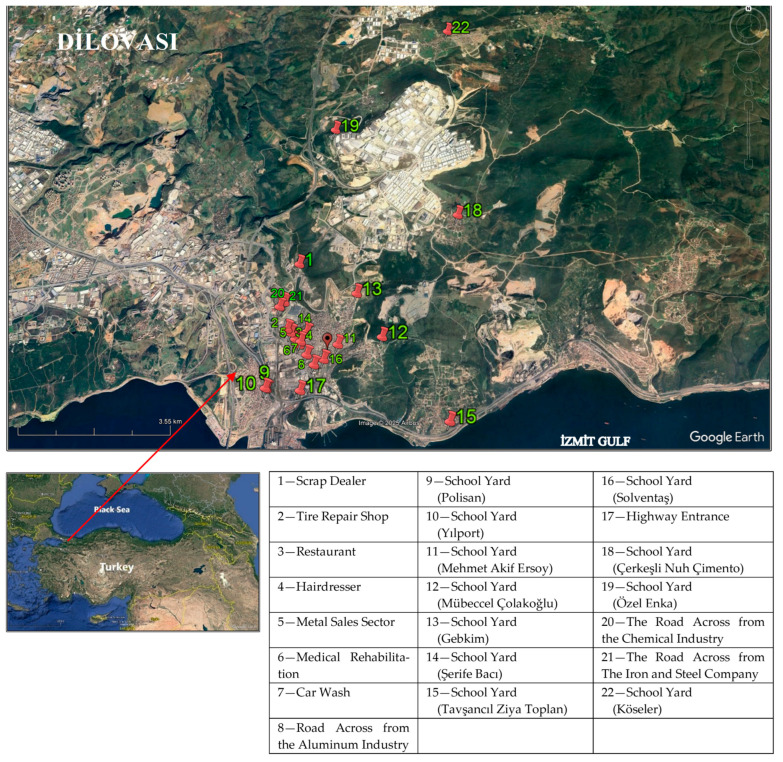
Study area [24].

This study presents the seasonal fluctuations, spatial distribution, and health risk assessment of the levels of 13 volatile organic compounds (VOCs), especially benzene, toluene, ethyl benzene, and xylene (BTEX), in the ambient air of Dilovası, which is affected by adverse environmental problems. Within the scope of the study, air samples were collected by passive tube sampling at twenty-two locations in Dilovası (Figure 1), and concentrations of the samples were determined by thermal desorption GC-MS. Golden Software Surfer program and QGIS Desktop 3.42.0 were used to create distribution maps based on the determined concentrations. Health risk assessment was performed using the methodology of the United States Environmental Protection Agency (USEPA). It is believed that this study, which assesses VOCs, particularly BTEX profiles, in this crucial settlement area linked to high cancer incidence rates, is significant in terms of highlighting these areas and bringing attention to the environmental issues caused by unplanned urbanization.

## 2. Materials and Methods

### 2.1. Sampling and Analysis

A sampling campaign was carried out in 22 locations in Dilovası province in 2022. The passive sampling procedure was applied for 7 days in the summer and 7 days in the winter period. Passive sampling tubes with tenax adsorbent were used for sampling. The TO-17 method of the US EPA was used for VOC sampling in this investigation [25]. Before sampling, the tubes were cleaned in a conditioning oven at 350 °C for 3 h. Following the conditioning process, the tubes were transported to the sample site by a refrigerated vehicle. Passive samplers were placed approximately 1.5 m above ground level. The sampling tubes completed their sampling in protective cages at the sampling points for 1 week. During the sampling process, diffusion covers were placed on the tubes to ensure that the right sample was collected. At the end of the sampling process, the caps of the tubes were closed and sent to the Bolu Abant Izzet Baysal University’s Scientific Industrial and Technological Applications and Research Center (SITARC) using a vehicle-type refrigerator. They were kept at −20 °C until analysis [25,26].

The collected samples were analyzed using a thermal desorber (TD; TD-100, Markes International Ltd., Llantrisant, UK), GC (Thermo Scientific 1300, Thermo Fisher Scientific Inc., Waltham, MA, USA), and MS (ISQ-QC, Thermo Fisher Scientific Inc., Waltham, MA, USA) operated in selected ion monitoring (SIM) mode. The column used was a fused-silica capillary column (TG-624; ID: 0.25 mm; length: 30 m; film: 1.4 μm), and the carrier gas was helium. The GC oven was programmed from 65 °C to 170 °C at a rate of 5 °C/min. After that, the temperature was raised by 10 °C/min to 220 °C and held isothermally at 220 °C for 5 min. The ion source temperature was 150 °C, the interface temperature was 230 °C, and the MS was conducted at 70 eV. The injection volume for analysis was 1 μL, and the detection limit was 0.1–0.5 μg/m^3^. Calibration was performed using the five-point external standard method. The National Institute of Standards and Technology (NIST) database, which has over 62,000 patterns, was used to interpret the GC-MS mass spectrum [26].

### 2.2. Distribution Maps

The coordinates of the sampling points in the study were taken from the UTM WGS84 Zone 35N system and then were processed on the map using the Golden Software Surfer program. The Triangulation with Linear Interpolation gridding method was used to transform the data corresponding to each point in the data file into a grid file. Then, the map image corresponding to the study area was taken from the OpenStreetMap map file, with coordinates, in the QGIS Desktop 3.42.0 software program. Ultimately, these two files were superimposed in the Surfer software program to create the final distribution maps.

### 2.3. Health Risk Assessment

A health risk assessment of VOCs was performed, considering both carcinogenic and non-carcinogenic effects. Among the VOCs studied, benzene, ethylbenzene, and naphthalene are classified as carcinogenic chemicals, while all VOCs except for n-propylbenzene, sec-butylbenzene, 4-isopropyl-toluene, and n-butylbenzene have non-carcinogenic effects. In the study, the assessment of the cancer and non-cancer health risks was carried out by adopting the inhalation pathway methodology of the United States Environmental Protection Agency (USEPA) [27]. The daily exposure (E) of an individual compound through inhalation was assessed by Equation (1):

E = C × IR × ED/BW(1)

where E is the daily exposure (mg/kg/day) and C is the pollutant concentration in air (mg/m^3^). Then the effective lifetime exposure (EL, mg/kg/day) was estimated from Equation (2).

EL = E × (D/7) × (WK/52) × YE/YL(2)

Exposure parameters used are given in Table 1. Considering the high mobility of people in this industrialized region, the exposure duration for adults was assumed to be 15 years based on field experience.

The integrated lifetime cancer risk (ILTCR) was calculated from Equation (3):

ILTCR = EL × CPF(3)

where CPF is the carcinogenic potency factor or cancer slope factor (1/(mg/kg/day)). The inhalation cancer slope factor of carcinogenic compounds was taken from the Risk Assessment Information System (RAIS) [28]. For the assessment, a cancer risk of >10^−6^ was considered as “carcinogenic effect of concern”, while a value <10^−6^ was considered as “acceptable level” based on USEPA’s approach [6,29]. The risk values between 10^−5^ and 10^−6^ have been declared as “acceptable” in some international and national guides [30,31], but since there are some studies suggesting possible carcinogenic risks from other chemicals in the Dilovası region [15], taking the threshold of 10^−6^ for the carcinogenic risk of VOCs could be more appropriate from a public health perspective.

On the other hand, the risk assessment for non-carcinogenic effects was expressed by hazard quotient (HQ), defined as the ratio of yearly average concentration (CAVG, mg/m^3^) to the reference concentration (RfC, mg/m^3^) calculated according to Equation (4).

HQ = CAVG/RfC(4)

RfC is defined as the inhalation reference concentration of the non-carcinogenic compounds, and RfCs of the compounds were obtained from the Integrated Risk Information System (IRIS) [28]. An HQ of >1 is considered as “adverse non-carcinogenic effect of concern”, while a value of HQ < 1 is considered as “acceptable level”. A summation of HQs for individual contaminants gives the Hazard Index (HI) [5,32].

All the exposure parameters, including CPF and RfC values, used in health risk assessment are tabulated in Table 1.

## 3. Results and Discussion

### 3.1. Concentration of VOCs in the Ambient Air

**Figure 2:**
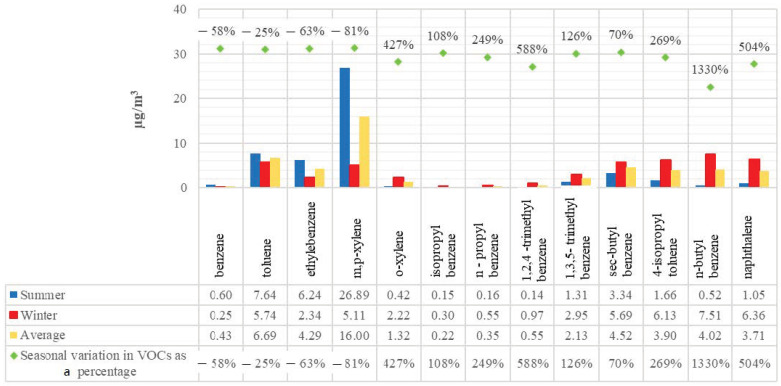
The mean concentrations of VOCs.

A total of 22 stations in Kocaeli Province’s Dilovası District monitored summer and winter VOC concentrations; the findings are shown in Figure 2. According to the averages of measurements taken at 22 stations, the concentrations of benzene, toluene, ethylbenzene, and m,p-xylene were found to be higher in the summer than in the winter (58%, 25%, 63%, and 81%, respectively), while other pollutants showed higher concentrations in the winter than in the summer. The slower rate of dissipation in wintertime could be the cause of may cause the greater amounts of VOCs other than BTEX that are observed during this season. While the average concentration of benzene, toluene, ethyl benzene and xylene total (ΣBTEX) in the region was determined as 41.79 µg/m^3^ in the summer, its average concentration in the winter was determined as 15.66 µg/m^3^. It is thought that the high ΣBTEX concentration in the summer may be due to the low decomposition rate. Additionally, traffic fluctuates based on the season. Summertime traffic in the city was heavier than the wintertime traffic [33]. By analyzing the measurements taken in Dilovası from 2014 to 2019, the study by Yılmaz et al. looked at the distribution of monthly vehicle counts. According to the study, there were more vehicles traveling through the area throughout the summer [34]. We think that traffic conditions in 2022 are comparable in scale and temporal behavior to the 2014–2019 years. Another factor that could contribute to a drop in BTEX concentration is the usual winter weather. Precipitation quickly spreads atmospheric BTEX to various environmental media like soil, plants, and roadbeds [35]. The climate in Dilovası is transitional between the Mediterranean and Black Sea climates, with rainy winters and hot summers. In Dilovası, the average yearly rainfall ranges from 768 mm to 1153 mm. October, November, and December have the highest rainfall. The average annual temperature is 14.5 °C. Winds in the region generally blow from the northeast [36].

Although limited research has shown that BTEX compound concentrations in the atmosphere are higher in the summer than in the winter, some places still showed higher levels in the winter. For instance, Mağat Türk’s study addressed the seasonal distribution of BTEX compounds in three distinct areas of Bolu’s city center: the roadside, the city center, and high-altitude settlements. The findings demonstrated that over the summer, BTEX compound concentrations rose over the summer, particularly in the village areas. The start of summer highland settlements and rising coal use were blamed for this surge [37]. In 2019, in Tehran, the health risk and disease burden caused by exposure to benzene, toluene, ethylbenzene, and xylene (BTEX) in outdoor air were assessed by Hosseini et al., based on data from five fixed stations with weekly BTEX measurements. The study revealed that the lowest seasonal BTEX concentrations occurred in the spring, while the highest concentrations occurred in the summer [38]. Khoshakhlagh et al. studied the summer and winter concentrations of BTEX pollutants in several workplaces at an oil refinery in Iran. The results of this study indicate that BTEX concentrations were higher in the summer than in the winter season for all workstations, especially for toluene and ethylbenzene [39]. Seasonal variation in VOCs is mainly controlled by the changes in mixing height, abundance of stationary and mobile sources, transport from neighboring states, photochemical loss from solar radiation, wet deposition and conversion to secondary organic aerosol [40]. Therefore, although the findings of the study were consistent with those of some articles [38,39], they contradicted others [5,6,40,41,42].

m,p-xylene was determined to be the most prevalent VOC among those evaluated in the research area (Figure 2). The average concentration in the summer was 26.89 µg/m^3^, and the average concentration in the winter was 5.11 µg/m^3^. The combustion process of engines and traffic is the main cause of the preponderance of xylene, as the majority of cars are powered by gasoline or diesel. As of 2024, the proportions of vehicles registered for use in Turkey were as follows: 34.1% diesel automobiles, 31.9% LPG cars, 30.2% gasoline-powered cars, 2.4% hybrid cars, and 1.1% electric cars. The percentage of cars with an unknown fuel type was 0.2%. Heavy commercial vehicles are fueled by diesel and liquefied natural gas (LNG) [43]. Diesel internal combustion engine emissions, evaporative emissions of gasoline, natural gas combustion, and vehicle exhaust are the largest contributors to ambient levels of VOCs [44]. According to the study by Srivastava et al., xylene is the primary species that differs between the exhaust of gasoline and diesel vehicles, with diesel exhaust being the primary source of xylene [45]. Dilovası, which was chosen as the study area, is one of the most important industrial areas in Kocaeli. It is located at a strategic point in the transition to Istanbul. It is on the route of the E-5 and Northern Marmara Motorway. Its status as a port city also makes it a frequent route for huge commercial vehicles. The daytime population increases due to thousands of employees coming to work in the industrial establishments in the district. Therefore, there is always heavy traffic in the district. This naturally makes xylene dominant. 

**Figure 3:**
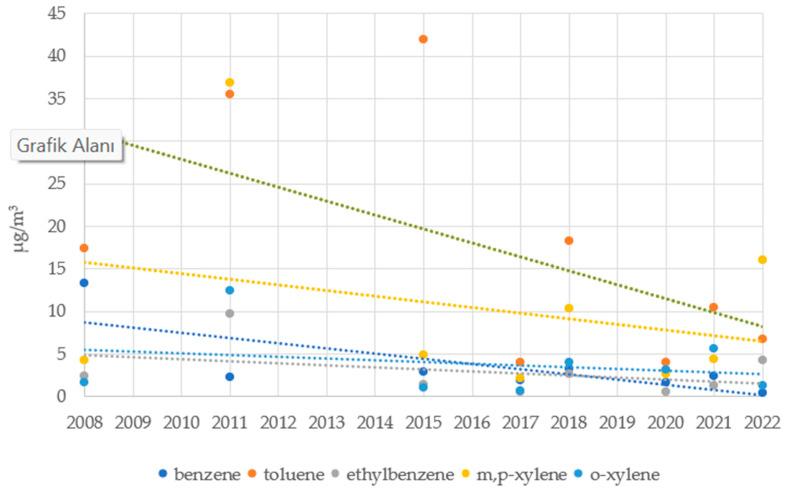
Comparison of VOC concentrations in the district as reported in this study and in previous years (Colored lines represent linear trend lines for each data group).

The study’s results were compared to those of other studies by Ergenekon et al. (2009), Pekey and Yılmaz (2011), and Pekey et al. (2015), which were carried out in the same district in the past, as well as with measurement results requested by an official letter from the Marmara Clean Air Center Directorate [19,22,46]. The results are shown in Figure 3. The ΣBTEX concentration, which was found to have an average of 28.72 µg/m^3^ in this study, was between 9.31 and 96.82 µg/m^3^ in prior years. When analyzed in terms of pollutant type dominance, Pekey and Yılmaz (2011) stated that xylene was a dominant pollutant, which aligns with the findings of this study [19]. According to research by Ergenekon et al. (2009) and Pekey et al. (2015), the concentration of toluene was found to be higher than that of other VOCs, indicating a particular level of VOC pollution [22,46]. It is an expected result that variations in concentration will occur based on the pollutant type and the year. The differences can be partly explained by differences in sampling strategy (sampling frequency, sampling period, and sampler type), but also by traffic, stationary emission sources, and meteorological conditions.

**Figure 4:**
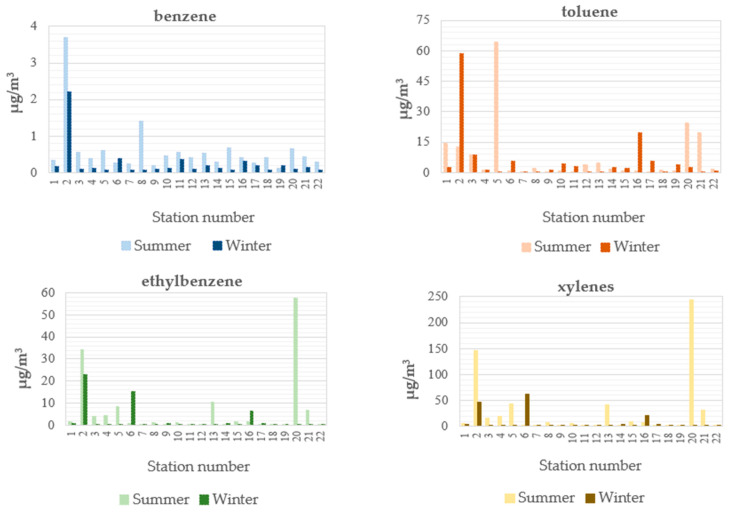
Concentrations of BTEX at the 22 sampling locations.

When the seasonal changes in BTEX concentrations were examined per station (Figure 4), it was observed that the seasonal changes in all compounds were not uniform. Although the average concentrations of benzene, toluene, ethylbenzene, and m,p-xylene, based on data from 22 stations, were found to be higher in the summer than in the winter (Figure 2), it was also observed that there were stations where concentrations were higher in the winter than in the summer. Specifically, benzene concentrations were higher at two measurement points (6 and 19); toluene at ten points (2, 6, 9, 10, 11, 14, 15, 16, 17, 19); ethyl benzene at six points (6, 9, 14, 16, 17, 19); and xylene at six points (6, 9, 14, 16, 17, 19).

Ratios between species were analyzed to compare BTEX emission sources, and Table 2 shows the findings. To differentiate solvent-related effects from vehicle-related effects, the toluene/benzene (T/B) ratio is used as a predictor. For example, a higher abundance of toluene is generally due to solvent usage and evaporation, while benzene indicates more fuel combustion [47]. A T/B ratio ranging from 1.5 to 4.3 indicates fuel combustion, while ratios outside this range are classified as other sources [38]. T/B ratios larger than 4.5 indicate the presence of non-traffic sources such as solvent vaporization, paint application, and industrial sources [48]. In particular, a T/B ratio exceeding 10 is considered a strong indicator of solvent effects [49]. Variations in T/B ratios are displayed in Table 1. While the highest T/B ratio was observed at the 5th sampling point with a value of 94.93 µg/m^3^, the lowest T/B ratio was observed at the 8th sampling point with a value of 1.52 µg/m^3^. T/B ratios between 1.5 and 4.3 were 23%, between those 4.3 and 10 were 31%, and those above 10 were 46%. The results demonstrated that industries dominated the district despite the substantial impact from traffic. The area is home to numerous ports, organized industrial zones, chemical, petroleum, rubber, iron and steel industries, and LPG storage tanks. It is situated in our nation’s main axis of industrial development. It is evident that VOC concentrations are affected by emissions from large, medium, and small industrial operations.

The xylene/ethylbenzene ratio (X/E) is another useful diagnostic index to estimate VOC sources. The ratio of the two compounds is frequently employed as an indicator of the photochemical reaction in the atmosphere because the rate at which OH radicals react with m/p/o-xylene is substantially faster than that of ethylbenzene. The value of X/E < 3 indicates a higher potential for m/p/o-xylene and OH radical reactions, a shorter VOC residence time in ambient air, and the presence of an aging air mass [49]. All X/E ratios that were determined within the scope of the study (Table 2) were found to be greater than 3, indicating the presence of fresh air pollution in the district.

Benzene/toluene (B/T) and xylene/benzene (X/B) ratios are also commonly used for the preliminary assessment of the air masses’ age and the determination of VOC sources, based on photochemical reactivity estimates. It has been suggested that the B/T ratio <0.4 and X/B ratio >1.1 indicate a young air parcel (local sources), while B/T ≥ 0.4 and X/B ≤ 1.1 indicate an old air parcel [49]. As seen in Table 1, 96% of the calculated B/T ratios were <0.4 and X/B ratios were >1.1, indicating that most of the air parcels in the district are young air masses. Therefore, industrial activities and transportation, including local vehicle exhaust, were considered one of the dominant VOC sources in the sampling area.

### 3.2. Spatial Analysis of BTEX and Naphthalene

**Figure 5:**
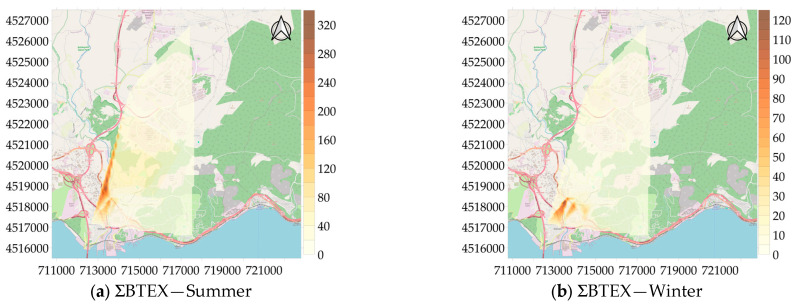
Spatial distribution of concentration of ΣBTEX (µg/m^3^).

Distribution maps were created for pollutants whose concentrations were determined at 22 measurement points and the distribution maps of ΣBTEX for the summer and winter seasons are given in Figure 5. Examining Figure 5, it is evident that the highly inhabited area where housing, industry, and traffic are linked has a higher concentration. In terms of ΣBTEX concentrations, the region where the 2nd measurement point, 5th measurement point, and 20th measurement point are located is particularly noteworthy. In the summer, the concentration reached 326 µg/m^3^ in this region and 130 µg/m^3^ in the winter. This area has heavy traffic, high-density buildings, and small industrial areas. The results can be attributed to these sources.

**Figure 6:**
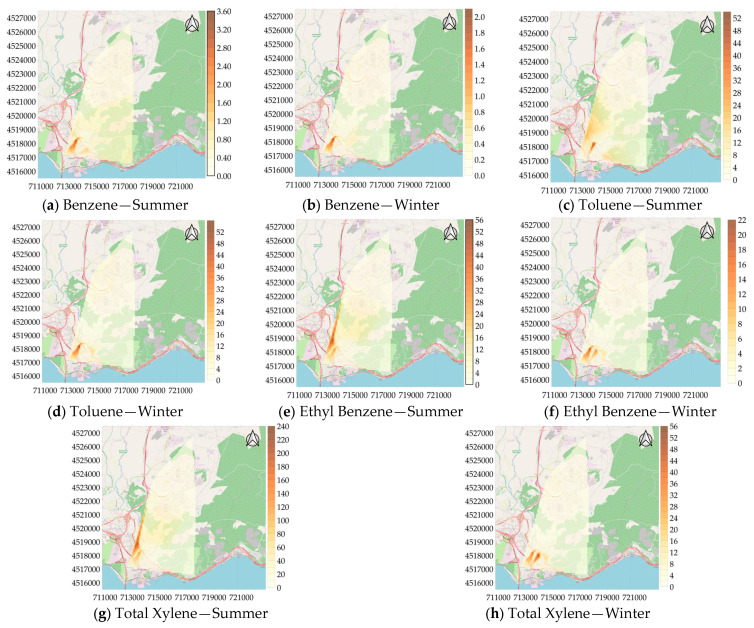
Spatial distribution of concentration of BTEX (µg/m^3^).

Individual BTEX compound distribution maps were made for the areas with higher pollution density compared to other areas, and these maps are shown in Figure 6. Upon examining the maps, it was discovered that the 2nd measurement point had the highest concentration of benzene during the summer, with 3.70 µg/m^3^, the 5th measurement point had the highest concentration of toluene, with 64.56 µg/m^3^, and the 20th measurement point had the highest concentrations of ethyl benzene and total xylene, at 57.42 µg/m^3^ and 243.76 µg/m^3^, respectively. In the winter, benzene was again found to be higher at the second measurement point with, 2.22 µg/m^3^, compared to the other measurement points. The concentrations of toluene and ethyl benzene were found to be higher at the second measurement point with concentrations of 58.67 µg/m^3^ and 22.84 µg/m^3^, compared to summer measurements. Total xylene was higher at the sixth measurement point with a concentration of 61.86 µg/m^3^, compared to other points. The results showed that, among the 20 measurement points, 4 measurement points in particular were characterized by high individual BTEX concentrations due to processes such as vehicle traffic, solvent use, painting processes, and thinner use.

According to the National Air Quality Assessment and Management Regulation, the annual average benzene concentration in ambient air should not exceed 5 μg/m^3^ [50]. The average benzene concentrations at the measurement stations were 0.60 µg/m^3^ for the summer and 0.25 µg/m^3^ for the winter. Since the average value was 0.43 µg/m^3^, no limit value exceedance was discovered when the measurement results were examined in this regard.

**Figure 7:**
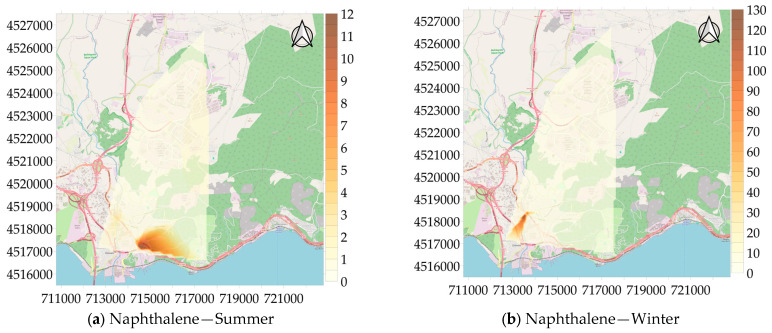
Spatial distribution of concentration of naphthalene (µg/m^3^).

Among the VOCs measured in the study area, distribution maps for naphthalene were created in addition to BTEX, and the maps are presented in Figure 7. When analyzing the maps, it can be seen that the first measurement point has the highest concentration of naphthalene in the summer (12.16 µg/m^3^), while the second measurement point shows the highest concentration in the winter (137.33 µg/m^3^). This seasonal variation in concentration is thought-provoking. Sources of naphthalene in open air mostly come from fugitive emissions and motor vehicle exhaust. Naphthalene is released into the atmosphere by evaporation, photolysis, adsorption, biodegradation, and land and water spills that occur during the storage, transportation, and disposal of coal tar and fuel oil [51]. As a result, the measurements of naphthalene conducted during this study point to fugitive emissions.

### 3.3. Health Risk Assessment of VOCs

**Figure 8:**
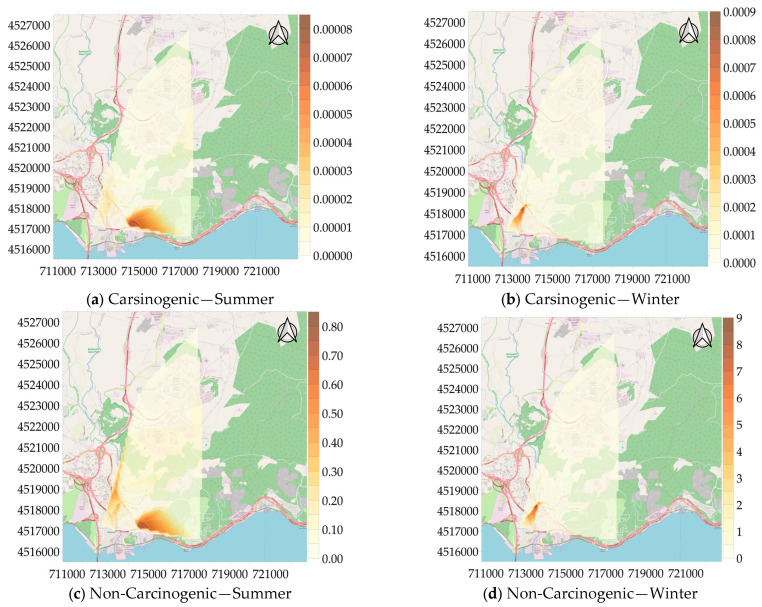
Carcinogenic and non-carcinogenic health risk assessment.

Health risk assessment for cancer and non-cancer risks of VOCs was carried out separately for each season seasonally. Health risk exposure distribution maps for benzene, ethylbenzene, and naphthalene, compounds classified as carcinogenic chemicals among the examined VOCs, and non-carcinogenic effects of all VOCs except n-propylbenzene, sec-butylbenzene, 4-isopropyl-toluene, and n-butylbenzene, are given in Figure 8. The carcinogenic risk (ILTCR) at 22 measurement stations was found to be between 6.7 × 10^−7^ and 8.3 × 10^5^ during the summer and between 1.8 × 10^−7^ and 9.4 × 10^−4^ during the winter. Especially in the summer, 19 out of 22 measurement points were above the carcinogenic risk (ILTCR) limit value of 1 × 10^−6^. The same was not true for the winter season. Overall, 9 out of the 22 measurement locations showed values over the limit value. However, in the winter period, the second measurement point attracted attention with its ILTCR value of 9.4 × 10^−4^. The findings indicate that residents of this area may be more susceptible to cancer if they are exposed to benzene, ethylbenzene, and naphthalene over an extended period of time. When the calculated non-carcinogenic risk (HQ) values were examined, the HQs ranged from 8 × 10^−3^ to 8.48 × 10^−1^ in the summer season. In the winter season, the values were between 3 × 10^−3^ and 1.3 × 10^−1^ at all measurement points except the second measurement point. Since it is less than 1, it was found that there is no significant risk of non-cancer health impacts at the other measurement points except for one measurement point in the study area. The HQ value at the second measurement site was determined to be 9.34. It is noteworthy that the naphthalene concentration reached 137.33 µg/m^3^ in the winter season at the second measurement point. When the sources of naphthalene compounds are examined, industrial activities and vehicle maintenance and repair workshops in the region stand out as important contributors [52]. Additionally, exhaust emissions from diesel vehicles are also an important source of naphthalene [53]. The naphthalene concentration at this measurement point, which is under the influence of these sources, was well above the RfC limit of 3 µg/m^3^ defined by the EPA. It is anticipated that exposure at this level could cause serious health effects. These findings are also supported by epidemiological studies examining occupational exposure. In a study conducted by Kamal and colleagues on auto mechanics and spray painters in Rawalpindi, Pakistan, it was found that blood naphthalene levels in these occupational groups were significantly higher compared to the control group. This indicates that occupational activities are a determining factor in naphthalene exposure [52].

The lipophilic nature of naphthalene allows it to be absorbed not only via inhalation but also through dermal and oral routes. Clinical evidence has shown that even dermal exposure alone can result in hematological disorders such as hemolytic anemia and methemoglobinemia. Additionally, oral exposure to naphthalene has been associated with toxic effects on the gastrointestinal system, liver, and kidneys, indicating a potential for multisystemic toxicity. Genetic predispositions may exacerbate these effects; for instance, individuals with glucose-6-phosphate dehydrogenase (G6PD) deficiency have been reported to exhibit more severe toxic responses to naphthalene. Chronic inhalational exposure is suggested to induce epithelial alterations in the upper respiratory tract, potentially initiating carcinogenic processes. However, epidemiological evidence directly connecting chronic naphthalene exposure to the development of cancer in human populations is still scarce, despite sufficient proof from animal models indicating the carcinogenic effects of naphthalene. Therefore, the International Agency for Research on Cancer (IARC) has classified naphthalene as Group 2B—possibly carcinogenic to humans, based on sufficient evidence from animal studies and limited evidence from human data [51]. In alignment with this assessment, Yost et al. (2021) conducted systematic evidence mapping and an evaluation of the existing toxicological and epidemiological data on naphthalene, with particular attention to its relevance for dose–response analysis and health risk characterization. Their review highlighted that while experimental animal studies provide robust data supporting respiratory tract toxicity and tumor formation, human studies often suffer from methodological constraints, such as short exposure durations, small sample sizes, and limited control for confounding variables, which weaken causal inference [54]. Taken together, these findings support the idea that exposure to naphthalene poses a risk to public health, especially in areas such as industrial sites and densely populated urban regions where exposure is likely to occur repeatedly, and they indicate the need for urgent control measures and routine monitoring, particularly in locations where this chemical is concentrated.

## 4. Conclusions

This study, which was carried out in one of Turkey’s most crucial regions, once again produced significant information regarding the region’s pollution The study’s findings demonstrated that the atmospheric VOC concentrations at the sampling locations varied significantly by season and location. In comparison to other measurement stations, higher concentrations were detected at stations 2, 5, 6, and 20 for the BTEX measurement and at station 2 for the naphthalene measurement. Upon closer inspection, it was found that these locations were surrounded by industrial activity and considerable traffic. In particular, there are industrial facilities that produce significant pollution, such as those that melt metal and scrap, manufacture paint and thinner, operate in the chemical and cosmetic industries, and recycle plastic. In addition, because of its closeness to ports, traffic on the E-5 and TEM highways is heavy. According to the findings of a carcinogenic risk assessment, long-term exposure to benzene, ethylbenzene, and naphthalene may raise the risk of cancer development in residents of this area. A non-carcinogenic risk (HQ) evaluation found that while there was no significant risk at 21 measurement points, there was a significant risk for non-cancer health effects at 1 measurement point (the second measurement point).

The study has once again demonstrated how crucial it is to implement better regulations and pollution control systems. Because Dilovası’s urban settlement grew haphazardly rather than according to plan, industry, traffic, and settlements have entwined and adversely impacted one another. Thorough research is important in this city and in other comparable cities that have emerged as examples of poor design. It is suggested that environmentally friendly industries be developed, traffic be controlled, industrial zones be reorganized, environmental quality and quality of life be improved, and an urban structure be established that guarantees the continuation of a healthy social life. Furthermore, the unique topographical characteristics of the Dilovası basin—situated within a narrow valley surrounded by hills—play a critical role in the accumulation and restricted dispersion of air pollutants. Due to this natural structure, the region tends to trap pollutants, thereby reducing the effectiveness of conventional emission control strategies. Therefore, it is recommended that topography be considered as an important factor in air quality management in regions such as Dilovası and integrated into pollution reduction planning.

Limitations of the study: This study solely includes measurements for the summer and winter seasons, with no data for the spring or autumn, day/night changes, or source distribution analysis. Increasing the frequency of VOC measurements in Dilovası, Turkey’s most polluted district, is important. Enhancing the frequency of measurements, particularly in locations classified as hot spots, and assessing health risks associated with BTEX exposure using a larger data set will be far more informative and useful for public health. In this study, only inhalation exposure was evaluated, not oral or dermal exposure (particularly for inhabitants in industrial districts). Future research should consider all possible exposure routes to naphthalene.

## Figures and Tables

**Table 1:** CPF and RfC values for VOCs and exposure parameters [28].

Compound	CPF (1/(mg/kg/day))	RfC (mg/m^3^)
benzene	2.73 × 10^−2^	0.03
toluene	NA	5
ethylbenzene	3.85 × 10^−3^	1
m,p-xylene	NA	0.1
o-xylene	NA	0.1
isopropylbenzene	NA	0.4
n-propylbenzene	NA	NA
1,2,4-trimethylbenzene	NA	0.2
1,3,5-trimethylbenzene	NA	0.2
sec-butylbenzene	NA	NA
4-isopropyl-toluene	NA	NA
n-butylbenzene	NA	NA
naphthalene	1.19 × 10^−1^	0.003
Exposure Parameters		
IR (inhalation rate for adults)		0.83 m^3^/h
ED (exposure duration for adults)		24 h/day
BW (body weight for adult)		70 kg
D (days per week exposure)		7 days
WK (weeks of exposure)		52 weeks
YE (years of exposure for adults)		15 years
YL (years in lifetime for adults)		75 years

“NA” (Not Available) values indicate that no reliable CPF or RfC data were available for the respective compounds in the RAIS, Toxicity Profile, 2010 database. This does not imply the compounds are safe, but rather that toxicity data were not provided by RAIS at that time.

**Table 2:** BTEX concentration ratios at different sampling locations.

Station Number	Average			
	T/B	X/E	B/T	X/B
1	33.15	4.62	0.03	20.48
2	12.04	3.37	0.08	32.53
3	26.31	3.96	0.04	26.12
4	5.68	4.30	0.18	39.51
5	**94.93**	5.23	0.01	65.16
6	9.82	4.05	0.10	96.17
7	3.14	4.40	0.32	8.19
8	**1.52**	5.83	0.66	5.63
9	6.13	5.75	0.16	20.23
10	8.70	4.52	0.11	12.12
11	3.81	5.45	0.26	**3.85**
12	8.70	5.30	0.11	6.02
13	7.56	4.12	0.13	60.63
14	10.84	4.78	0.09	12.60
15	3.96	5.75	0.25	12.81
16	27.60	3.49	0.04	38.04
17	12.80	4.66	0.08	13.80
18	3.80	4.77	0.26	6.18
19	13.54	5.25	0.07	13.03
20	34.37	4.25	0.03	**314.85**
21	33.68	4.55	0.03	53.52
22	7.54	5.32	0.13	10.29

The values indicated in bold are the max and min values.

## Data Availability

All data used in the creation of tables and figures are available from the corresponding author upon request.

## Abbreviations

The following abbreviations are used in this manuscript:

BTEX	Benzene, Toluene, Ethyl Benzene, And Xylene
CPF	The Carcinogenic Potency Factor Or Cancer Slope Factor
HAPs	Hazardous Air Pollutants
HI	Hazard Index
HQ	Hazard Quotient
IARC	The International Agency for Research on Cancer
ILTCR	Integrated Lifetime Cancer Risk
LNG	Liquefied Natural Gas
NIST	The National Institute of Standards and Technology
RAIS	Risk Assessment Information System
RfC	Reference Concentration
SITARC	Scientific Industrial and Technological Applications and Research Center
US EPA	U.S. Environmental Protection Agency
VOCs	Volatile Organic Compounds
